# Robust arterial compliance estimation with Katz’s fractal dimension of photoplethysmography

**DOI:** 10.3389/fphys.2024.1398904

**Published:** 2024-06-10

**Authors:** Xiaoman Xing, Jingyuan Hong, Jordi Alastruey, Xi Long, Haipeng Liu, Wen-Fei Dong

**Affiliations:** ^1^ School of Biomedical Engineering (Suzhou), Division of Life Sciences and Medicine, University of Science and Technology of China, Suzhou, China; ^2^ Suzhou Institute of Biomedical Engineering and Technology, Chinese Academy of Sciences, Suzhou, China; ^3^ Division of Imaging Sciences and Biomedical Engineering, King’s College London, St. Thomas’ Hospital, London, United Kingdom; ^4^ Department of Electrical Engineering, Eindhoven University of Technology, Eindhoven, Netherlands; ^5^ Centre for Intelligent Healthcare, Coventry University, Coventry, United Kingdom

**Keywords:** arterial compliance, Katz’s fractal dimension, photoplethysmography, noise-resistance, single-site

## Abstract

Arterial compliance (AC) plays a crucial role in vascular aging and cardiovascular disease. The ability to continuously estimate aortic AC or its surrogate, pulse pressure (PP), through wearable devices is highly desirable, given its strong association with daily activities. While the single-site photoplethysmography (PPG)-derived arterial stiffness indices show reasonable correlations with AC, they are susceptible to noise interference, limiting their practical use. To overcome this challenge, our study introduces a noise-resistant indicator of AC: Katz’s fractal dimension (KFD) of PPG signals. We showed that KFD integrated the signal complexity arising from compliance changes across a cardiac cycle and vascular structural complexity, thereby decreasing its dependence on individual characteristic points. To assess its capability in measuring AC, we conducted a comprehensive evaluation using both *in silico* studies with 4374 virtual human data and real-world measurements. In the virtual human studies, KFD demonstrated a strong correlation with AC (*r* = 0.75), which only experienced a slight decrease to 0.66 at a signal-to-noise ratio of 15dB, surpassing the best PPG-morphology-derived AC measure (*r* = 0.41) under the same noise condition. In addition, we observed that KFD’s sensitivity to AC varied based on the individual’s hemodynamic status, which may further enhance the accuracy of AC estimations. These *in silico* findings were supported by real-world measurements encompassing diverse health conditions. In conclusion, our study suggests that PPG-derived KFD has the potential to continuously and reliably monitor arterial compliance, enabling unobtrusive and wearable assessment of cardiovascular health.

## 1 Introduction

Arterial compliance (AC) has significant value when managing cardiovascular disease, as it is closely linked to cardiovascular events and all-cause mortality (Vlachopoulos et al., 2010). Clinical guidelines often recommend the use of carotid-femoral pulse wave velocity (cfPWV) measured by applanation tonometer as a reliable measure of overall vascular compliance for cardiovascular risk stratification (Laurent et al., 2006; Mancia et al., 2013; Pereira et al., 2013; Pereira et al., 2015). Aortic pulse wave velocity (aoPWV) measured through magnetic resonance imaging (MRI) is also utilized as an independent predictor (Voges et al., 2012). However, these measurements require skilled operators, limiting their widespread application. Pulse pressure (PP), on the other hand, is a relatively easy measurement that reflects AC. It serves as a robust indicator for calcified atherosclerosis in various vascular beds (Bortel et al., 2001; Niiranen et al., 2019). Nevertheless, PP measured using the oscillometric method provides only intermittent evaluation of the vascular condition and does not capture the dynamic changes that occur throughout the day due to factors like temperature, physical activity, and stress.

Photoplethysmography (PPG) offers the advantage of continuity, allowing for long-term study and correlation with health outcomes. However, the performance of PPG-derived surrogate AC indices, such as the stiffness index (SI_ppg_), aging index (AGI), and reflection index (RI), heavily rely on the quality of PPG morphology and characteristic points, which are susceptible to degradation with added noise (Pannier et al., 2002; Charlton et al., 2021; Hong et al., 2023). On the other hand, PPG-derived AC measures by machine learning techniques, such as convolutional neural networks and long-short-term memory networks, often utilize the complete waveform, temporal correlation or chaotic features to predict cardiovascular functions (Radha et al., 2019; Khalid et al., 2020; Schlesinger et al., 2020; Mejía-Mejía et al., 2021; Khodabakhshi et al., 2022; Wang et al., 2022). While these methods demonstrate good performance in specific datasets, their internal mechanisms remain unknown, which may limit their ability to generalize to populations with different hemodynamic statuses. Nevertheless, these algorithms and relevant studies identified the temporal fluctuation or complexity of PPG as a promising candidate for AC assessment (Sviridova et al., 2018; Xing et al., 2023a; Xing et al., 2023b).

In recent years, the concept of fractal dimension (FD), a widely used mathematical tool in biomedical signal processing, has shown promise in AC estimation (Sviridova and Sakai, 2015; Sviridova et al., 2018; Xing et al., 2023a). FD captures the geometric complexity of signals and encodes transient changes of physiological status into the temporal patterns. For example, Esteller *et al.* found that Katz’s FD (KFD) yielded consistent results in discriminating between states of brain function and was less susceptible to noise effects (Esteller et al., 2001). In our previous comprehensive search, we found that Higuchi FD is closely related to mean blood pressure (BP), while KFD is significantly associated with compliance (Xing et al., 2023a; Xing et al., 2023b). It is important to note that the compliance estimation method employed in those studies was imprecise and did not undergo validation against a gold standard. Despite the demonstrated utility of FD in estimating hemodynamics, the physiological meaning or origin of FD remains unclear (Khodabakhshi et al., 2022). Sviridova *et al.* proposed that the chaotic characteristics of PPG could arise from a set of differential equations, reminiscent of those incorporated in the four-element Windkessel model, which considers pressure, volume, flow, and employs principles of momentum conservation (Sviridova and Sakai, 2015; Wang et al., 2017). While Windkessel models offer valuable insights into the interplay between KFD and various hemodynamic parameters, such as compliance, they inherently fail to fully account for the complex multiple wave reflections occurring within the intricately branching vasculature, thereby emphasizing the requirement for a deeper, more sophisticated understanding.

To address these concerns, we propose conducting *in silico* studies (with synthetized virtual data) using the more advanced one-dimensional model and known hemodynamic parameters, alongside the experimental study (with real human data), to compare and validate our proposal. The aim is to build a framework for a physiological explanation of complexity measures such as KFD and thoroughly evaluate its potential benefits in estimating AC.

## 2 Materials and methods

### 2.1 Data source

#### 2.1.1 *In silico* databases

In order to explore the potential role of KFD as a surrogate measure of AC, this study employed both *in silico* and experimental data. The *in silico* data were generated using three different models with similar distribution of hemodynamic properties to form three databases.

**FIGURE 1:**
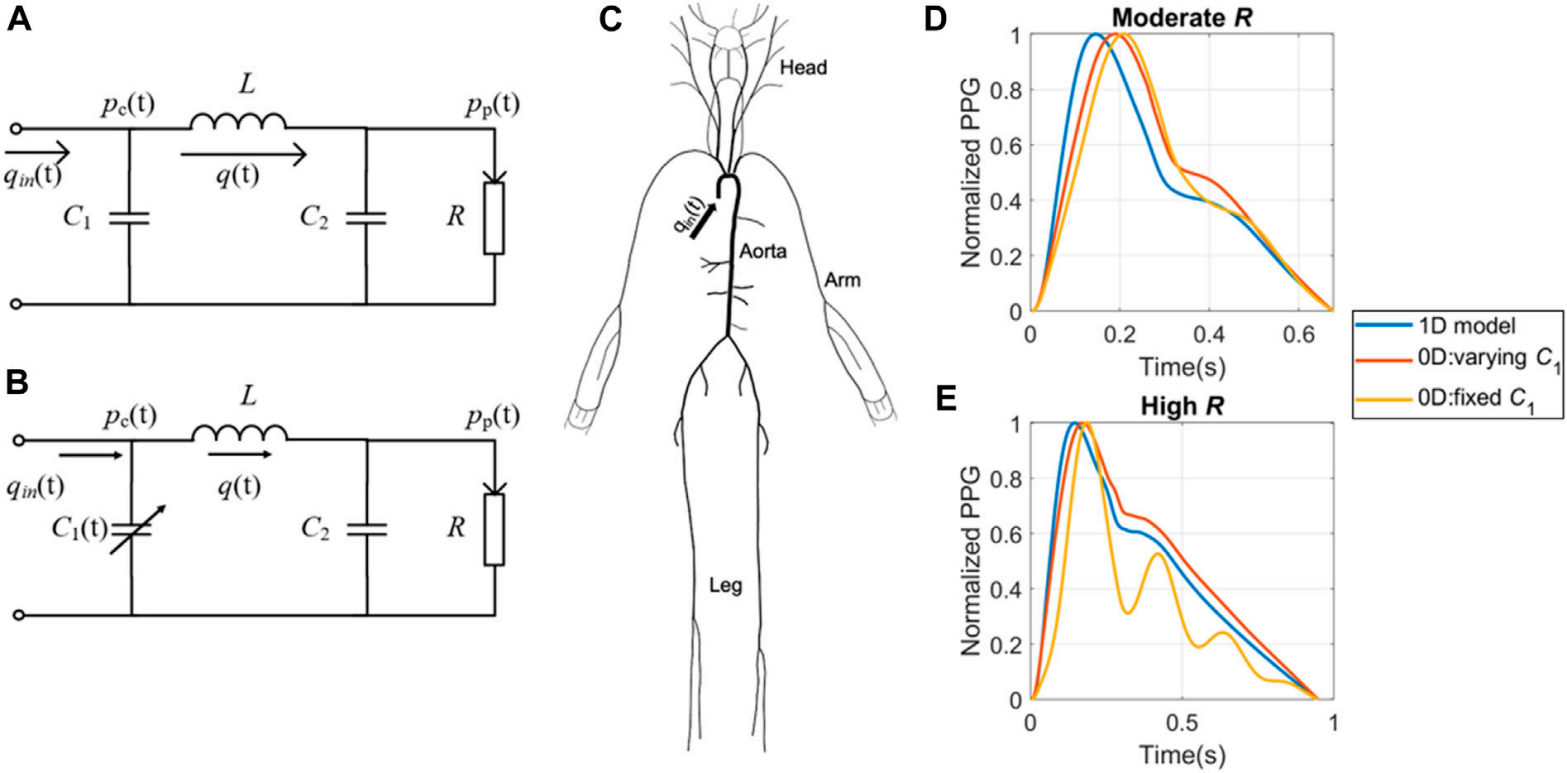
Simulation models used to create the *in silico* datasets. **(A)** 0D model with fixed central compliance (*C*_1_). **(B)** 0D model with time-dependent *C*_1_. **(C)** 1D model with distributed compliances, resistances and blood flow. Examples of waveforms generated by the three theoretical models at **(D)** moderate and **(E)** high peripheral resistance respectively. *p*_c_(t) refers to central BP; *p*_p_(t) refers to peripheral BP; *q*_in_(t) refers to the cardiac flow waveform during a cardiac cycle; *q*(t) refers to vascular blood flow; *C*_2_ refers to peripheral compliance; *L* refers to inertance; *R* refers to peripheral resistance.

The first *in silico* database consists of simulated waveforms of BP and PPG for a single cardiac cycle from various arteries (aorta, head, neck, torso, and limbs) in 4374 virtual subjects spanning six age decades (25–75 years old). We hereby refer to this database as PWDB (Charlton et al., 2021; Hong et al., 2023). Among these subjects, 537 exhibited BPs outside of healthy ranges. This was predominantly due to abnormal aortic or brachial PP (observed in 431 subjects) and abnormally high ratio of brachial to aortic PP (90 of the remainder) (Charlton et al., 2019). However, we still included these subjects in our analysis. This choice was based on the fact that these subjects still adhere to model-based physiological rules, and may occur in scenarios such as surgeries (Lee et al., 2022). This database employed a one-dimensional (1D) blood flow model to incorporate different cardiovascular parameters from 116 systemic arterial segments. A simplified illustration is shown in Figure 1C. In previous studies utilizing PWDB, the aortic Young’s modulus (*E*_Ao_) has been commonly employed to evaluate vascular aging. However, when it comes to BP or PP estimation, AC may hold greater relevance. AC is inversely proportional to Young’s modulus and represents the ratio of blood volumetric changes to PP. In our study, we primarily utilized AC as the reference and examined its correlation with PP and temporal complexity measures of PPG. To determine the correlations between the estimated parameters and the reference, we employed the Pearson correlation coefficient (PCC).

The second and third *in silico* databases were generated using four-element Windkessel (WK4) models, as illustrated in Figures 1A, B. These models are zero-dimensional (0D) lumped models that do not consider segmented pulse wave reflections (Segers et al., 2008; Xing et al., 2023b). In the classic 0D model, the central compliance (*C*_1_) is assumed to be fixed (Segers et al., 2008), as exemplified in Equation Set 1, which shares similarities with the equations governing the chaotic Rössler system (Sviridova and Sakai, 2015). In reality, *C*_1_ varies throughout the cardiac cycle, and this variation can result in the formation of temporal complexity patterns (Xing et al., 2023b). To capture the dynamics of varying *C*_1_, we developed a modified WK4 model, as presented in Eq. 2. This adaptation involved treating *C*_1_ as a temporally evolving variable and designating *p*_p_(t) as the sole unknown parameter.

$$\left\{\begin{array}{c} \frac{dq\left(t\right)}{dt}=\frac{1}{L}\left(p_{c}\left(t\right)-p_{p}\left(t\right)\right)\ldots \ldots \ldots \left(1a\right)\\ \frac{dp_{c}\left(t\right)}{dt}=\frac{1}{C_{1}}\left(q_{in}\left(t\right)-q\left(t\right)\right)\ldots \ldots \ldots \left(1b\right)\\ \frac{dp_{p}\left(t\right)}{dt}=\frac{1}{C_{2}}\left(q\left(t\right)-\frac{p_{p}\left(t\right)}{R}\right)\ldots \ldots \ldots \left(1c\right) \end{array}\right.$$

$$\frac{d^{3}p_{p}\left(t\right)}{dt^{3}}+\frac{1}{RC_{2}}\frac{d^{2}p_{p}\left(t\right)}{dt^{2}}+\left(\frac{1}{LC_{1}\left(t\right)}+\frac{1}{LC_{2}}\right)\frac{dp_{p}\left(t\right)}{dt}+\frac{1}{LRC_{1}\left(t\right)C_{2}}p_{p}\left(t\right)=\frac{1}{LC_{1}\left(t\right)C_{2}}q_{\text{in}}\left(t\right)$$ (2)

In these equations, *p*_c_(t) is the central BP, *p*_p_(t) is the peripheral BP, *R* is the peripheral resistance (PVR), *C*_2_ is the peripheral compliance (PVC), and *L* is the inertance. *q*(t) refers to blood flow and *q*_in_(t) refers to the cardiac flow waveform during a cardiac cycle. To ensure a fair comparison, we employed hemodynamic profiles that closely resembled those of the 4374 virtual subjects in the PWDB. By generating an equivalent number of virtual measurements, we aimed to maintain consistency throughout. The detailed 0D model construction procedures are described in the Appendix.

By employing these three models, we can investigate some enduring questions regarding the origin of the temporal complexity of PPG. Specifically, we hypothesize that if temporal complexity patterns arise from frequency-dependent PPG wave transmission, both WK4 databases should demonstrate a significant correlation between KFD and AC. If temporal complexity patterns are only observed in WK4 models with varying *C*_1_ and PWDB, it implies that the temporal variation during a cardiac cycle aids in the formation of complexity. Conversely, if the hemodynamics-induced temporal patterns are exclusively observed in the 1D model-generated PPG, it suggests that signal complexity originates from back-reflected waves caused by multiple junctions during wave propagation. Importantly, both compliance variations and reflected waves can coexist and contribute to the observed complexity in physiological signals. However, it is worth mentioning that this analysis does not encompass longer-term complexity (>1 cardiac cycle), such as that observed in closed-loop models or with beat-to-beat cardiac output (CO) fluctuations.

Despite our efforts to align the key hemodynamic parameters, certain discrepancies remain evident. At low to moderate *R*, the two 0D models exhibited a close resemblance to each other and the 1D model, as shown in Figure 1D. However, as *R* increases to high *R*, the 0D model with fixed *C*_1_ displays significant distortions, as shown in Figure 1E.

#### 2.1.2 Experiment databases

The study collected experimental data from two publicly available sources, exhibiting varying levels of noise and targeting different populations. The first source (Dataset1) utilized was a short-recorded PPG dataset for BP monitoring (Liang et al., 2018). This dataset contains PPG and BP data from 219 subjects with 657 measurements in a sitting position, covering an age range of 20–89 years and including individuals with hypertension and diabetes. Each subject underwent three measurements, each lasting 2.1 s, with the collection of reference BP from the opposite arm shortly before the PPG measurement. Data acquisition was performed using the SEP9AF-2 PPG sensor (SMPLUS Company, Korea), positioned on the left fingertip. The sensor utilized dual LEDs with wavelengths of 660 nm and 905 nm, a sampling rate of 1 kHz, and a 12-bit ADC. Signal processing involved a 0.5–12 Hz bandpass filter.

**FIGURE 2:**
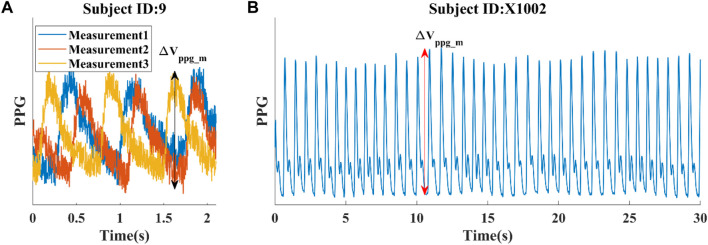
Illustrations of data from two experimental datasets. **(A)** Dataset 1: Three short PPG measurements obtained from a single subject, with a reference SBP/DBP of 123/73 mmHg taken shortly before the PPG measurement at the opposite arm. **(B)** Dataset 2: First 30 s of a continuous PPG measurement. Average SBP/DBP for this subject is 122/68 mmHg. The pulsatile amplitude of PPG (ΔV_ppg_m_) depends on the device, tissue coupling coefficient, and pulsatile blood volume (ΔV_ppg_).

The second database (Dataset2) used in the study was created by Carlson *et al.* from Kansas State University (Carlson C. et al., 2020). It consists of data collected from 40 healthy subjects in a supine position for approximately 5 min. A vital sign monitor (GE Datex Ohmeda CardioCap 5, USA) was used to gather finger PPGs with a sampling rate of 100 Hz. The output PPG was internally filtered by a lowpass filtered with a cutoff frequency of 10 Hz. The reference BP was obtained by a beat-to-beat BP monitor (Finometer PRO, Finapres Medical Systems, the Netherlands) at the finger site and reconstructed to brachial BP. This dataset includes parameters such as stroke volume (SV), BP, PPG, and more. Table 1 shows a comprehensive overview of participants’ information, and Figure 2 showcases representative data from the two datasets. To compare the distribution of characteristics of the datasets, we employed a Student’s t-test with uneven sample sizes.

**TABLE 1:** Characteristics of the real-world datasets.

	Dataset1	Dataset2
Subjects	219	40
Posture	Sitting	Supine
Sex (F/M)	115/104	23/17
Measurements	657	40
Duration (s)	2.1	∼300
Reference BP	Discrete	Continuous
Age (years)	58 ± 16*	34 ± 15
Height (cm)	160 ± 8*	171 ± 11
Weight (kg)	60 ± 12*	76 ± 18
SBP (mmHg)	126 ± 20*	146 ± 25
DBP (mmHg)	70 ± 11*	88 ± 18

*Significantly different between groups (*p* < 0.05).

BP. blood pressure; SBP. systolic blood pressure; DBP. diastolic blood pressure; F. female; M: male.

### 2.2 Signal pre-processing procedure

**FIGURE 3:**
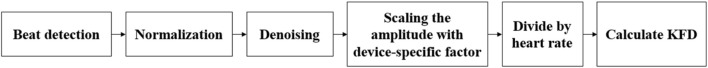
Optimal pre-processing steps.

The original PPG signals, especially PPG from Dataset 1 is very noisy, hindering its ability to extract morphological features, and fractal dimensional information. For optimal data preprocessing, we recommend following the steps outlined in Figure 3. Firstly, it is crucial to accurately identify each heartbeat. The benchmark Multi-Scale Peak and Trough Detection algorithm is recommended (Bishop and Ercole, 2018). Secondly, normalization should be applied to obtain a standardized PPG waveform with a maximum height of one and a minimum of zero. To reduce noise and restore some of the distortions, denoising technique should be applied. Additionally, scaling the amplitude of the waveform with a device-specific factor, determined by the sampling rate, is recommended. The detailed explanation of the rationale behind this scaling procedure can be found in Section 2.3. Finally, dividing by the heart rate and calculating KFD will allow for robust and meaningful information extraction. By following these steps, we obtained reliable results in the analysis.

To find the optimal scaling factor and denoising technique that best preserves the AC information, we compared different scaling strategies and several popular filtering techniques, which are introduced in Section 2.3 and Section 2.5.

The normalization of PPG waveforms relies on accurately estimating the pulsatile amplitude of PPG (ΔV_ppg_), which measures blood volume at the fingertip in our study. For *in silico* data, we calculated ΔV_ppg_ by multiplying the pulsatile cross-sectional vascular area with the vascular length. To maintain simplicity while preserving the validity of the conclusion, we assumed a vascular length of 1 cm at the measurement site. For experimental data, the pulsatile portion of PPG (ΔV_ppg_m_) was obtained by calculating the difference between detected peaks and troughs of each cardiac cycle, as illustrated in Figure 2. It is important to note that the ΔV_ppg_m_ does not provide an absolute measure of blood volume. Instead, it also depends on the device and tissue coupling coefficient, which requires personalized calibration.

In this study, our primary focus was on utilizing SI_ppg_ as a comparative measure due to its strong correlation with *E*_Ao_ in virtual human data (Hong et al., 2023). Additionally, we calculated RI and AGI to assess their susceptibility to noise interference. To ensure precise and reliable feature estimation, we leveraged the code published by Charlton *et al.* and Hong *et al.* (Charlton et al., 2019; Hong et al., 2023). We made minor adjustments to address the issue of failure when applied to experimental data. The signal quality was assessed following the methodology outlined by Orphanidou *et al.* (Orphanidou et al., 2015), utilizing a template-matching approach. Measurements that did not meet the predefined quality threshold were excluded from the analysis.

### 2.3 Katz’s fractal dimension

Compared to SI_ppg_, which relies on characteristic points and is vulnerable to noise, KFD is derived from the entire waveform, making it robust against noise. The definition of KFD is as in Eq. 3.

$$\text{KFD}=\frac{\log_{10}L}{\log_{10}d}$$ (3)

where *L* is the sum of distances between successive points, and *d* is the estimate of diameter between the first point of the sequence (*1*_st_) and the point with the farthest distance among all other points (*i*th). Mathematically, *d* can be expressed as

$$d=\max\left(\text{distance}\left(1_{st},i_{th}\right)\right)$$ (4)

In this context, the term “distance” refers to the Euclidean distance between the *i*th point of the sequence and the first point. Although KFD is a dimensionless measure of the distance ratio, it still exhibits a reliance on the signal’s amplitude. This reliance arises from the fact that the distance calculation comprises two dimensions: time and amplitude. The choice of time and amplitude scales plays an important role in determining the relative importance of these dimensions in the distance calculation. Additionally, the duration of the cardiac cycle varies, resulting in a variable length of the time axis.

To address these issues, we firstly normalized the PPG amplitude and adjusted the time axis using the heart rate. Subsequently, we determined an optimal universal scaling factor *α* that allowed us to maximize the correlation between KFD and AC. We conducted tests by using a scaling factor of *α×*F_s_/HR, where *α* was chosen to vary from 0.1 to 0.5 with a step size of 0.05. Here, HR represents the heart rate in units of Hz, and F_s_ denotes the sampling rate. This approach ensured that the two axes shared the same unit and had a comparable scale. We applied the same scaling factor to all *in silico* models. For comparing the three models, we employed a Student’s t-test with equal sample sizes. To compare the correlations between KFD and AC with PPG morphology-derived indices and AC, we computed the confidence intervals of the correlation coefficients. If the confidence intervals do not overlap, we interpret the correlation coefficients as significantly different.

We also utilized a probability-based outlier detection algorithm to clean KFD data, similar to the method proposed by McCool *et al.* (McCool et al., 2016). Initially, the KFD was calculated without explicitly checking the data quality. Subsequently, the state transition probability was determined for discretized KFD values. If the probability of KFD state transition fell below 1% of the whole dataset, the corresponding states related to the KFD were labeled as outliers. This approach allowed us to identify and exclude potential outliers in the data, ensuring the accuracy and reliability of the results. Data points outside the range of the median plus or minus three times the standard deviation (SD) were also excluded. We followed a similar procedure to remove outliers of AC.

### 2.4 Estimation of AC in experimental setting

When working with *in silico* data, hemodynamic parameters like AC are known. However, accurately quantifying absolute compliance for *in vivo* data posed challenges without advanced MRI scanning or applanation tonometry. In this study, we utilized PP as an intermediate variable to assess the correlation between KFD and AC.

PP, which is measured in the experimental data, has long been used as an indicator of AC, expressed as AC_est_ = *f* (PP). In this study, for easier comparison between *in silico* and *in vivo* data, we also investigated the correlation between PP and KFD, represented as PP_est_ = *g* (KFD). By showcasing the robust reversibility of the functions *f* (PP) and *g* (KFD), we can conclude that KFD can serve as a reliable indicator of AC. If these functions are able to withstand noise and disturbances, then the estimation of AC_est_ and PP_est_ can be considered robust.

To assess the function *f* (PP), we examined the relationship between AC, KFD and several PPG-morphology-derived features, as well as their combinations. Similarly, we obtained *g* (KFD).

### 2.5 Influence of noise and filtering

Representative factors influencing PPG signals encompass motion artifacts, powerline interference, low amplitude, and premature ventricular contraction (Elgendi, 2012). These factors can be identified, eliminated, or rectified (Elgendi, 2016; Shin, 2022). The remaining noise is predominantly white Gaussian (Tang et al., 2020). Noise has a strong impact, not only on SI_ppg_, but also on KFD. To obtain comparable results from signals with different levels of noise, we firstly have to design a filter to restore the original PPG signals, which conform to physiological mechanisms. In this process, the filter with the least loss of AC information should be chosen.

To investigate the noise effect, white Gaussian noise was added to the *in silico* PPG signals (PWDB) to simulate instrumental noise (Redheuil et al., 2010). Noise was superimposed on the simulated data and a bandpass filter (Kaiser window FIR filter, 0.665–35 Hz) was used to remove the noise. As reported by Hong et al. (2023), three levels of noise intensity were generated by setting signal-to-noise ratio (SNR) to 15, 20, and 30 dB. We replicated these noise generation and filtering parameters to facilitate direct comparisons with the aforementioned study. The performance of SI_ppg_ and KFD with and without noise was compared.

For real human data, it was observed that certain signals exhibited noticeable levels of noise and corruption, which could not be effectively eliminated by the bandpass filters (Charlton et al., 2022). As the experimental signal underwent processing by the internal filter, the original noise type and level became unknown. Nonetheless, residual noise remains prominent in Dataset1. Therefore, we measured the residual noise in Dataset1 to gauge the relative noise level. The noise was calculated as the variance between the noisy signal and the further filtered data, with the filtered signal designated as the clean signal. Three signal filtering techniques were implemented and compared: median smoothing (Li et al., 2018), bandpass filtering (Hong et al., 2023), and knowledge-based multi-Gaussian fitting (Banerjee et al., 2015). The correlations of SI_ppg_, RI, AGI, and KFD with AC were assessed using the most effective filter. To mitigate the impact of outliers, SI_ppg_ beyond a standard deviation from the median was excluded. RI and AGI, having a lower standard deviation, had the threshold set at two standard deviations. The outlier detection approach for KFD was elaborated in section 2.3.

For median smoothing, a sliding window of 15 data points was used to smooth out the signal. The window size was empirically selected and was suitable for both experimental datasets, despite the difference in sampling rates. This technique aids in mitigating abrupt fluctuations and noise in the waveform.

For the bandpass filtering, we employed the identical bandpass filter utilized in the evaluation of noise effects in the *in silico* PWDB (Hong et al., 2023). While Dataset1 and Dataset2 underwent preprocessing with bandpass and lowpass filters, respectively, the specific filter designs used in these processes were unknown. Our study revealed that incorporating an additional Kaiser window FIR filter resulted in further signal improvement.

The knowledge-based multi-Gaussian fitting technique assumes that PPG waveforms are composed of five Gaussian waveforms representing the forward and back reflected waves (Couceiro et al., 2015). Rules are applied to restrict the amplitude and arrival time of each Gaussian wave. Specifically, we postulate the presence of two forward waves, along with three waves involving one or multiple reflections. In accordance with this model, single-reflected waves exhibit diminished amplitudes compared to the principal forward waves, with subsequent reflections displaying even smaller intensities due to progressive energy dissipation. The arrival sequence of reflected waves is consistent with a progression from single-reflection to double-reflection, and ultimately to multiple-reflections. More details could be found in the previous work (Couceiro et al., 2015). The resulting fitted waveform serves as a denoised representation based on expected PPG waveform characteristics. We evaluated the effect of these filters on the correlation of SI_ppg_, KFD, AC, and PP. The filter that best preserved the intercorrelation between these parameters, leading to a minimal decline in correlation coefficients in the presence of noise, was chosen as the most suitable filter.

### 2.6 Sensitivity analysis

The analysis of KFD sensitivity to AC is crucial. For instance, in areas where sensitivity is reduced, it is recommended to refrain from solely relying on KFD for quantitative AC estimation. Nevertheless, leveraging the collective sensitivity patterns of various features can lead to a more reliable and robust AC estimation.

A straight line has a fractal dimension of 1. As the simulated PPG signals in our study were smooth and free from measurement errors, the resulting KFD value was slightly above 1, exhibiting a narrow dynamic range. To highlight the pulsatile or “fractal portion” of the curvature, it is advisable to subtract the baseline one from the fractal dimension. Thus, we adjusted the relative sensitivity index (*I*) originally proposed by Hong *et al.* (Hong et al., 2023), by choosing a divisor of KFD-1.

For PWDB data, when an individual model input parameter was independently varied by one SD from its baseline value, we calculated the individual sensitivity of KFD to these hemodynamic changes as follows:

$$I_{individual}=\frac{V-V_{0}}{\left(V_{0}-1\right)v}\times 100$$ (5)

$V_{0}\text{ and }V$ represent, respectively, the baseline and the value in response to perturbation, and *v* indicates variations (±SD) for each input parameter (Charlton et al., 2019). Theses input parameters includes SV, AC, and PVR. To analyze the distribution of *I*_individual_ across different hemodynamic statuses, we recorded the *I*_individual_ and three key parameters: AC, *R*, and CO at the baseline. For ease of comparison, we selected *I*_individual_ of SI_ppg_ for analysis. RI and AGI could be examined in a similar way.

In our analysis of the experimental data, we aimed to determine the sensitivity of KFD to each variable while considering potential confounding factors. To achieve this, we calculated the partial correlation between KFD and each parameter. Since only Dataset2 provided continuous recordings with variations, we exclusively used Dataset2 for the sensitivity analysis. Within Dataset2, we had access to measurements of SV and BP. Consequently, we substituted CO and AC with SV and PP as the key parameters. To estimate *R*, we divided the mean blood pressure (MAP) by CO (Westerhof et al., 2019). It is worth noting that, we used ∂KFD/∂PP^−1^ to estimate the sensitivity of KFD to AC.

## 3 Results

The results were presented in the following order. First, surrogate AC measures were proposed and evaluated using virtual human data. Next, the study optimized KFD calculation procedure to maximize its correlation with AC. The potential origin of KFD was also investigated by comparing three theoretical models of varying complexity. The robustness and sensitivity of KFD to AC was tested using virtual human data to establish the theoretical limits. Finally, using real human subjects of different age groups and health conditions, the study verified the findings from *in silico* studies and showed potential limitations of the method.

### 3.1 Estimation of AC

In the PWDB dataset, a direct correlation between AC and KFD could be readily established. However, when dealing with real-world human data lacking readily accessible AC values, an indirect approach relying on PP as an intermediary parameter becomes necessary. To establish connections between AC and PP, and then between PP and KFD, we constructed a sequential linkage among these variables, allowing us to infer the predictability of AC through this indirect chain of associations.

**FIGURE 4:**
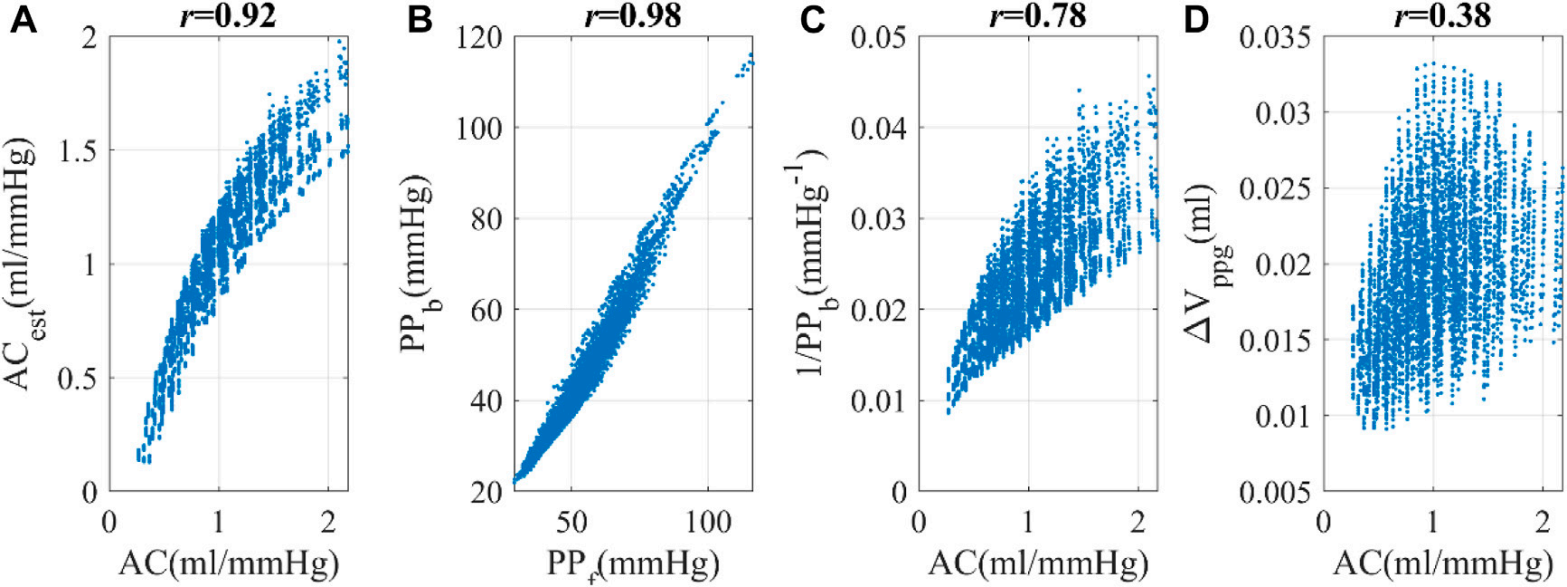
Simulated hemodynamic parameters (from the PWDB data) **(A)** Correlation between arterial compliance (AC) and estimated arterial compliance (AC_est_), a linear combination of ΔV_ppg_ and PP^−1^. **(B)** Correlation between PP_f_ and PP_b_, the pulse pressure at the finger and brachial locations respectively. **(C)** Correlation between AC and 1/PP_b_. **(D)** Correlation between AC and ΔV_ppg_, the pulsatile blood volume at the peripheral site.

With regards to the correlation between AC and PP, we discovered that a linear combination of 1/PP_b_, with PP_b_ as the pulse pressure at the brachial location, and finger pulsatile blood volume (ΔV_ppg_) exhibits a strong correlation with AC (*r* = 0.92), as shown in Eq. 6 and Figure 4A. Eq. 6 is an explicit expression of *f* (PP) described in the method section. In this context, *a* and *b* serve as constants, further substantiating the notion that PP can serve as an effective surrogate for AC in instances where direct AC measurements are unavailable.

$${\text{AC}}_{\text{est}}=a\times \Delta V_{\text{ppg}}+b/\text{PP }∼f\left(\text{PP}\right)$$ (6)

ΔV_ppg_, though susceptible to variations stemming from elements like hydration levels, non-hematological cellular constituents, cutaneous pigmentation, contact force, and individual-specific calibration parameters, tends to exhibit a commendable degree of consistency within a given subject due to the relative constancy of these influencing factors. The robust correlation of 0.78 observed between 1/PP and AC underscores the pivotal role of PP in determining AC_est_. Therefore, Eq. 6 represented by *f* (PP) should be resilient against uncertainties introduced by ΔV_ppg_m_. In the PWDB dataset, the numeric expression of *f* (PP) became AC_est_ = −0.98 + 40.56ΔV_ppg_+51.62/PP, wherein the variables ΔV_ppg_ and PP are quantified with respective units of “milliliters (mL)" and “millimeters of mercury (mmHg)". Based on this knowledge, we utilized 1/PP_b_ and its linear combination with ΔV_ppg_ as an alternative indicator of arterial compliance, referred to as AC_est_. While it would be logical to use the pulse pressure at the finger site (PP_f_), this hemodynamic quantity is not commonly measured. As PP_b_ demonstrates a linear correlation with PP_f_, we opted to use PP_b_ in our subsequent investigations, as shown in Figure 4B. For the sake of clarity, we will refer to PP_b_ as PP throughout this study.

### 3.2 Optimization of KFD calculation and its *In silico* correlation with AC

**FIGURE 5:**
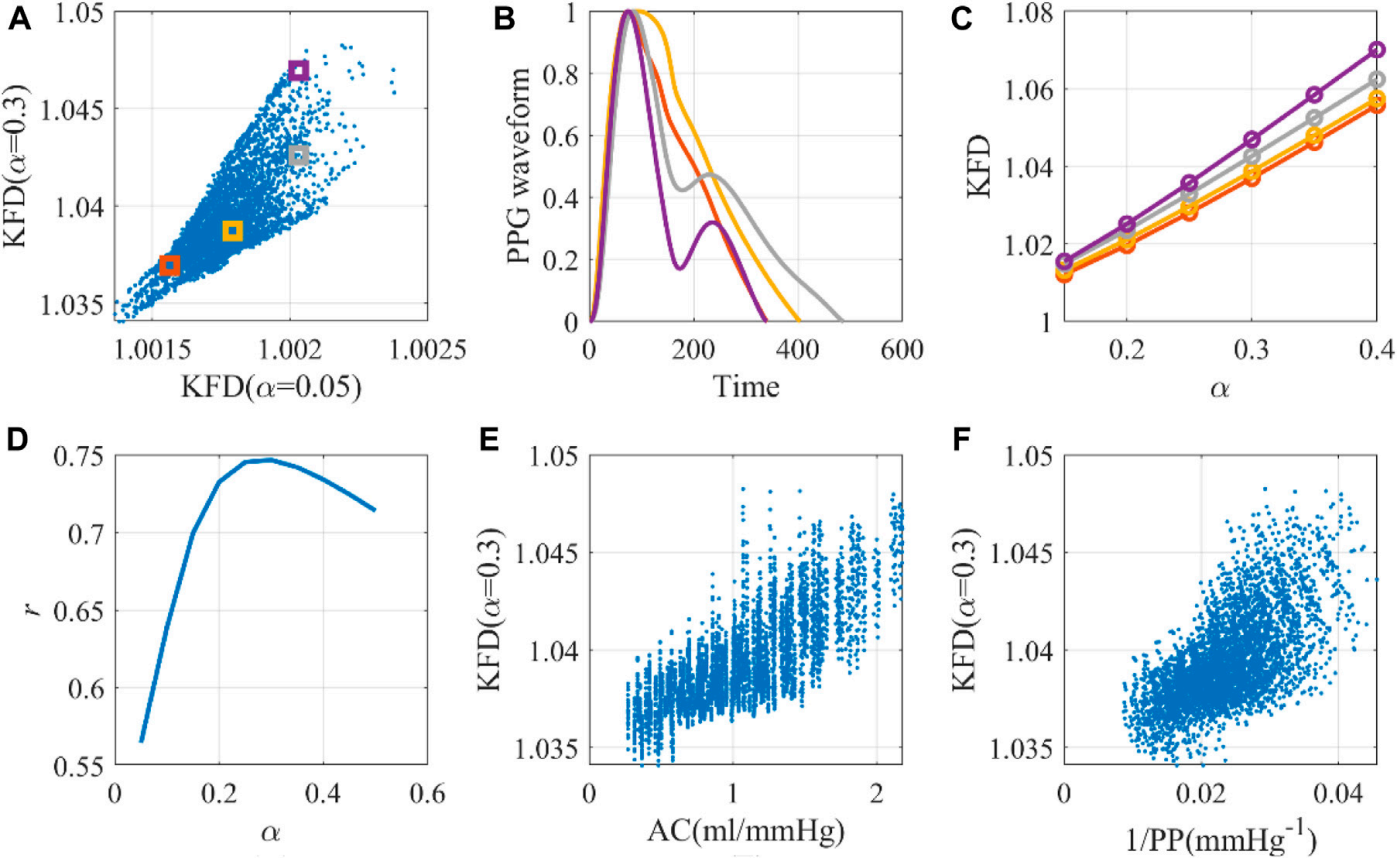
The optimal universal scaling factor for KFD calculation with a single cardiac cycle. **(A)** Scale-dependence of KFD, with selected virtual human data for detailed investigation. **(B)** Waveforms corresponding to selected virtual human data. **(C)** KFD changes caused by scaling factor α. **(D)** Optimal *α* for KFD calculation. **(E)** The AC-KFD correlation with the optimal scaling factor: 0.3 (*r* = 0.75). **(F)** The 1/PP-KFD correlation with the optimal scaling factor (*r* = 0.65).

In Figure 5A, we observed that KFD’s overall relationship with α is non-linear. The optimal α value was determined to be 0.3 (*r* = 0.75), as evidenced in Figures 5D, E. Using this scheme, the PPG axis was scaled to an average of 0.3 s, which is slightly lower than half the average duration of the cardiac cycle, which is 0.82 s. The variation in KFD in response to differing waveforms and α values is visually depicted in Figures 5B, C. Considering that the trace of PPG waveform is more than twice the peak amplitude, it is a reasonable scaling factor. Using the correlation between KFD and *E*_Ao_ as a target led to the same optimal scaling factor *α.* However, it is noteworthy that the best correlation between KFD and *E*_Ao_ was relatively smaller (*r* = −0.58). If we use PP^−1^ as the reference, its best correlation with KFD is 0.65, as shown in Figure 5F. Combining KFD and ΔV_ppg_ linearly only marginally improved this correlation to 0.72. This relationship is represented by Eq. 7, which is an explicit expression of *g* (KFD) described in the method section. Here, *c* and *d* are constants.

$$1/{\text{PP}}_{\text{est}}=c\times \Delta V_{\text{ppg}}+d\times \text{KFD}∼g\left(\text{KFD}\right)$$ (7)

As such, in the PWDB dataset, the specific numeric form of *g* (KFD) assumes the following expression: 1/PP_est_ = −2.05-0.42ΔV_ppg_+2.01KFD. Combining with Eq. 6, we can examine whether KFD might function as a predictor of PP and, subsequently, an indicator of AC, as shown in Eq. 8.

$$\text{KFD}\propto \frac{1}{d\times \text{PP}}-\frac{c}{d}\times \Delta V_{\text{ppg}}∼{\text{AC}}_{\text{est}}$$ (8)

In PWDB, the explicit dependence of AC_est_ on KFD was expressed as AC_est_ = 107.04 + 18.81ΔV_ppg_-103.61KFD.

**FIGURE 6:**
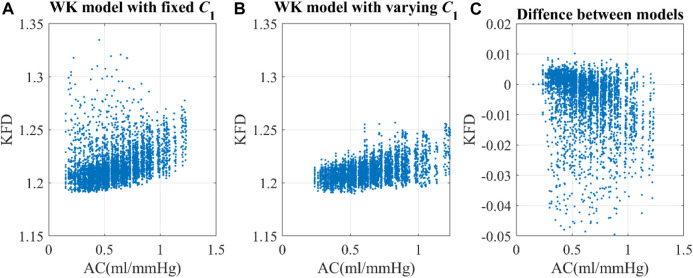
Correlation of arterial compliance and KFD using **(A)** WK model with fixed *C*_1_ and **(B)** WK model with varying *C*_1_. **(C)** The difference between models were calculated by subtracting KFD with fixed *C*_1_ from KFD with varying *C*_1_.

Utilizing the same optimal scaling factor, the WK4 models with fixed *C*_1_ and time-dependent *C*_1_ displayed correlations of 0.19 and 0.49, respectively, between KFD and AC, as shown in Figure 6. One interesting observation is that the KFD has fewer outliers and showed a better linear correlation with AC when *C*_1_ is time-dependent. We did not perform a correlation analysis with *E*_Ao_, as its role in the 0D model was not clearly defined.

### 3.3 Effect of data quality and noise using *In silico* data (PWDB)

**FIGURE 7:**
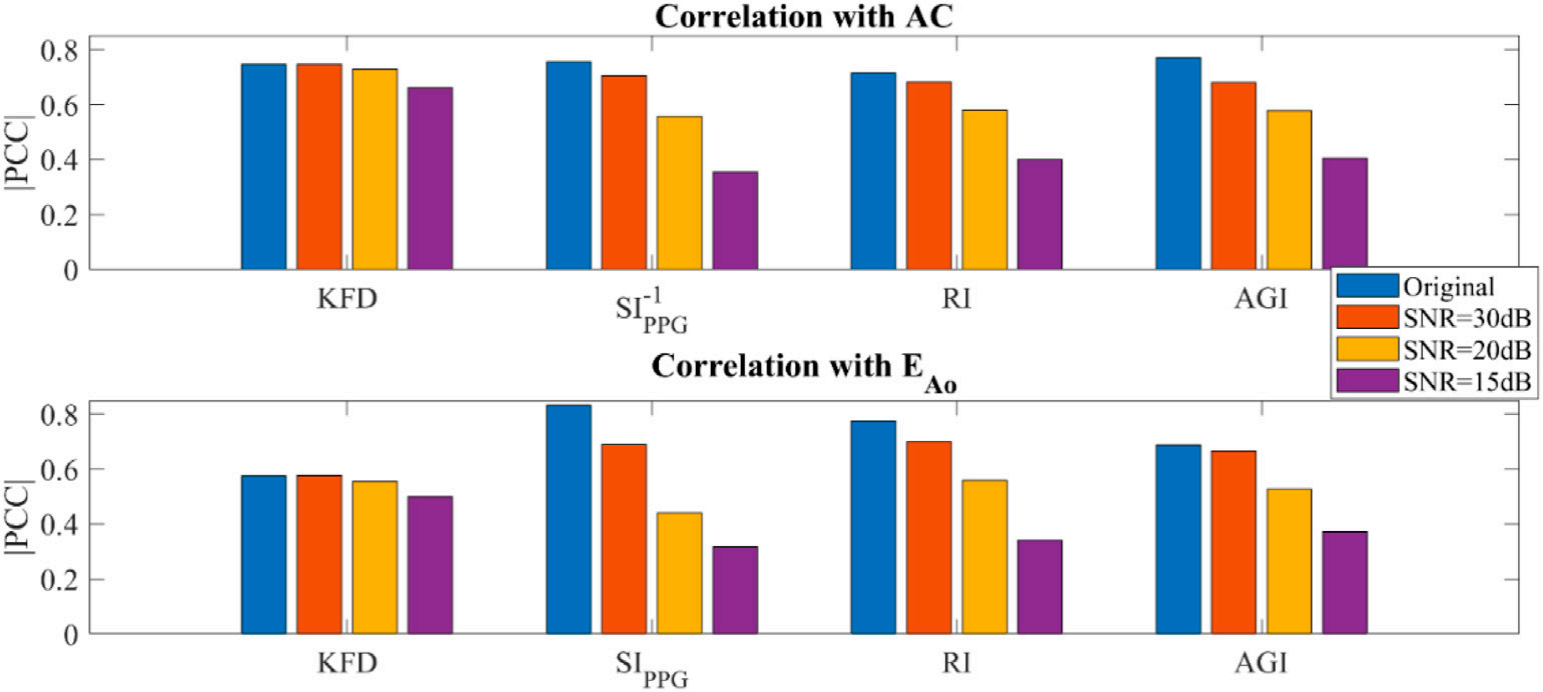
Simulated hemodynamic parameters with added noise and filtering (PWDB). **(A)** Correlation of KFD, SI_ppg_^-1^, RI, AGI and AC. **(B)** Correlation of KFD, SI_ppg_, RI, AGI and *E*_Ao_.

In PWDB (*in silico* data) with artificially added noise, as the SNR levels decreased, we observed an increase in fluctuations in the PPG morphologies for all virtual subjects, which could not be completely eliminated with bandpass filters. When examining morphology-derived features like SI_ppg_, RI and AGI, we found a significant decrease in their correlation with AC as the noise level increased. Notably, SI_ppg_ exhibits a nonlinear association with AC, prompting the utilization of SI_ppg_^-1^ for calculating its correlation with AC. In contrast, the correlation between KFD and AC or *E*_Ao_ was minimally affected by the noise level, as shown in Figure 7. It is important to note that these conclusions only hold true when filters are employed. KFD relies on waveform trace measurement, and increased noise significantly affects KFD values and reduces the correlation.

### 3.4 Sensitivity of KFD to hemodynamic parameters using *In silico* data (PWDB)

**FIGURE 8:**
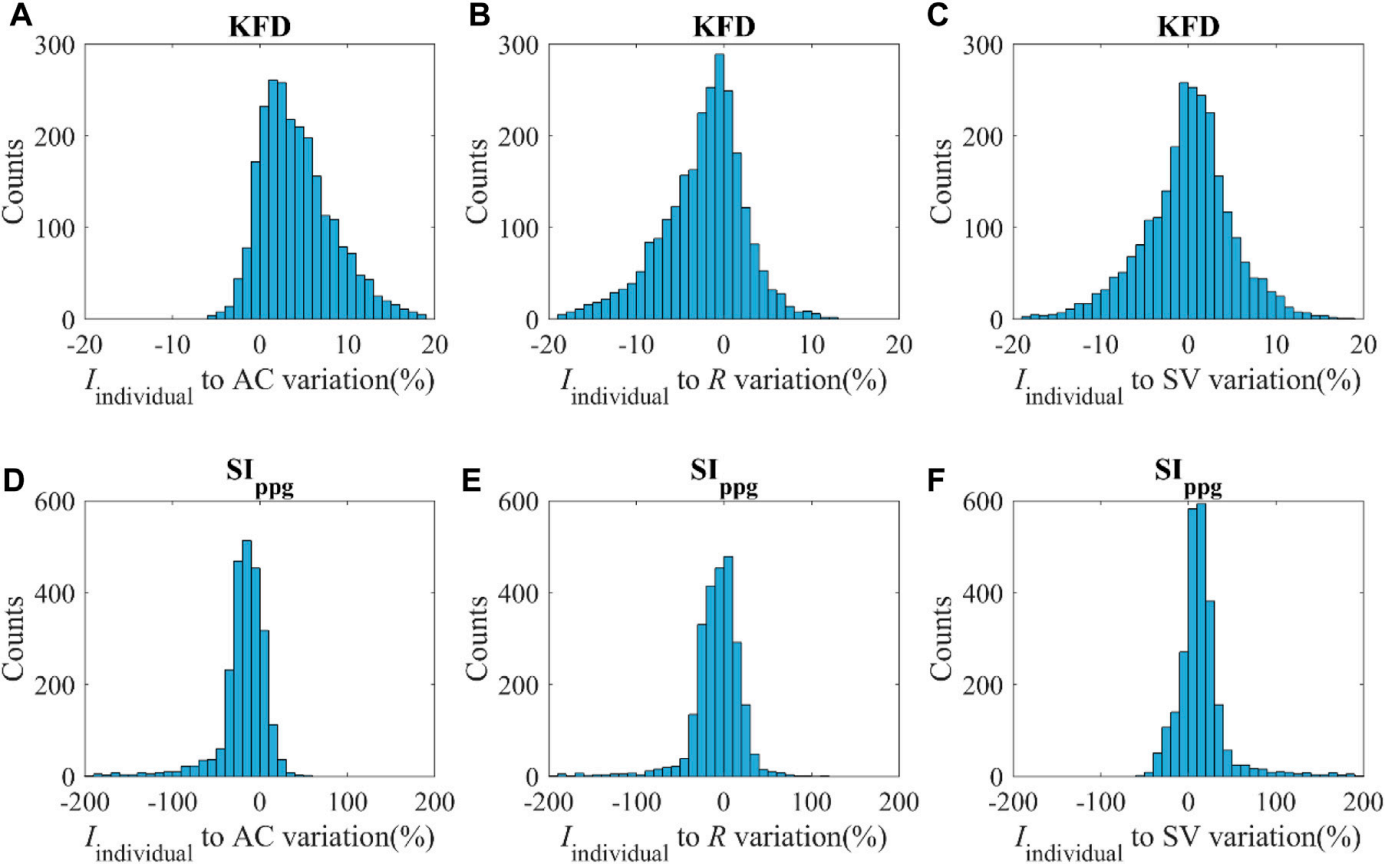
**(A–C)** Distribution of the *I*_individual_ of KFD in response to variations of AC, *R* and SV with the magnitude of *v*. **(D–F)** Distribution of *I*_individual_ of SI_ppg_ in response to variations of AC, *R* and SV with the magnitude of *v*; *v* indicates ±SD of AC, *R* and SV respectively.

KFD is primarily influenced by AC, PVR, and SV. However, it is important to note that individuals exhibit distinct behaviors during different hemodynamic processes, as shown in Figure 8. For instance, when only AC undergoes changes while other hemodynamic parameters remain constant, the *I*_individual_ of KFD tends to be mostly positive, with reduced sensitivity in certain AC regions. This behavior is inherent to the hemodynamic process and not a result of experimental noise. Similarly, when PVR changes, the *I*_individual_ of KFD predominantly shows negative values, with diminishing sensitivity observed at certain hemodynamic statuses. Similar mixed sign patterns were observed in *I*_individual_ of SI_ppg_, with a few instances of particularly high sensitivities.

We compared the hemodynamic status of both negative and positive *I*_individual_ of KFD and SI_ppg_, as shown in Table 2. Our analysis revealed significant differences in certain hemodynamic parameters. This finding presents challenges when attempting to use a single index to track intra-subject AC, *R*, or SV, as the sensitivity can be either positive or negative due to the unknown hemodynamic status or the source of fluctuation in real experiments.

**TABLE 2:** Hemodynamic status of *in silico* simulation with positive and negative *I*_individual_.

	*v*	Positive *I*_individual_				Negative *I*_individual_			
		N (%)	SV (mL)	AC (mL/mmHg)	*R* (mmHg.s/mL)	N (%)	SV (mL)	AC (mL/mmHg)	*R* (mmHg.s/mL)
KFD	ΔAC	87	61.7 ± 4.1*	1.03 ± 0.26*	0.78 ± 0.07*	13	59.4 ± 3.7*	0.88 ± 0.23*	0.82 ± 0.06*
	Δ*R*	31	61.7 ± 4.1	1.03 ± 0.25	0.78 ± 0.07	69	61.7 ± 4.1	1.03 ± 0.25	0.78 ± 0.07
	ΔSV	53	59.4 ± 4.1*	0.88 ± 0.25*	0.82 ± 0.07*	47	61.7 ± 3.9*	1.03 ± 0.25*	0.78 ± 0.07*
SI_ppg_	ΔAC	20	59.4 ± 3.1	0.88 ± 0.19*	0.82 ± 0.05*	80	61.7 ± 4.1	1.03 ± 0.26*	0.78 ± 0.07*
	Δ*R*	61	61.7 ± 4.1	1.03 ± 0.26	0.78 ± 0.07	39	61.7 ± 4.1	1.03 ± 0.25	0.78 ± 0.07
	ΔSV	24	64.3 ± 4.1*	1.23 ± 0.26*	0.72 ± 0.07*	76	59.4 ± 4.0*	0.88 ± 0.23*	0.82 ± 0.07*

N, percentage of positive or negative *i*_individual_ in virtual human data; * indicate a significant difference between the hemodynamic parameters in the positive or negative *I*_individual_ group. A Student’s t-test with uneven sample sizes was used.

However, this finding can also be advantageous if appropriate maneuvers are employed to clarify ambiguous predictions about the hemodynamic status. For example, if a known maneuver is performed to increase SV, and we observe an increase in KFD and decrease in SI_ppg_, it is highly likely that the subject had a lower baseline AC initially. This possibility of a chain reaction may help improve long-term, ambulatory AC measurements.

### 3.5 Experimental PP and KFD correlation

**FIGURE 9:**
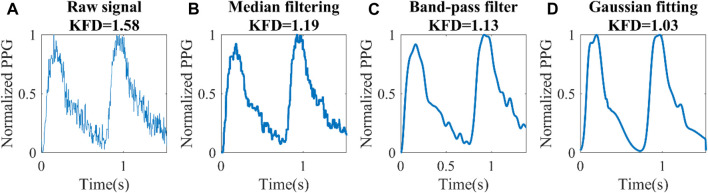
Different filtering techniques for a set of experimental data (Dataset1). **(A)** Raw signal, **(B)** Median filter, **(C)** Band-pass filter, **(D)** Knowledge-based multi-Gaussian filtering.

The original SNR of both datasets remains undisclosed. Following internal filtering of the devices, the median residual SNR is 21.4 dB in Dataset1 and varies from 8.2 dB to 31.7 dB. In Dataset2, the median residual SNR is 31.8 dB, varying from 6.1 dB to 37.5 dB. This noise presents a notable challenge since both SI_ppg_ and KFD calculations necessitate high-quality data. Figure 9 illustrates how residual waveform distortion can lead to artificially inflated KFD values that do not reflect hemodynamics accurately. To address this issue, we tested and compared three filtering techniques: median filter, band-pass filter and multi-Gaussian fitting.

The distortion of the waveform had a significant effect on KFD. Consequently, achieving a reasonable overall waveform recovery becomes crucial. In Dataset 2, the Gaussian filter yielded a correlation of 0.58 between KFD and 1/PP. However, when employing bandpass and median filters, the correlation dropped to 0.14 and 0.15, respectively. For Dataset1, due to significantly higher noise level, the correlation coefficients were 0.36, 0.05, and 0.16 for Gaussian filtering, band-pass and median filters, respectively. Hence, Gaussian filtering emerged as the most effective method for preserving PP-related information. It is important to mention that two subjects were excluded from Dataset2 due to obesity, with body mass index (BMI) of 48 and 36 respectively. We observed that these two subjects have artificially high KFD. This can be attributed to the impact of excessive contact pressure on the finger, which alters transmural pressure and local compliance, in turn disrupting the estimation of central arterial compliance. Another five subjects were excluded due to low ΔV_ppg_m_ (<0.05), which indicates poor perfusion.

**FIGURE 10:**
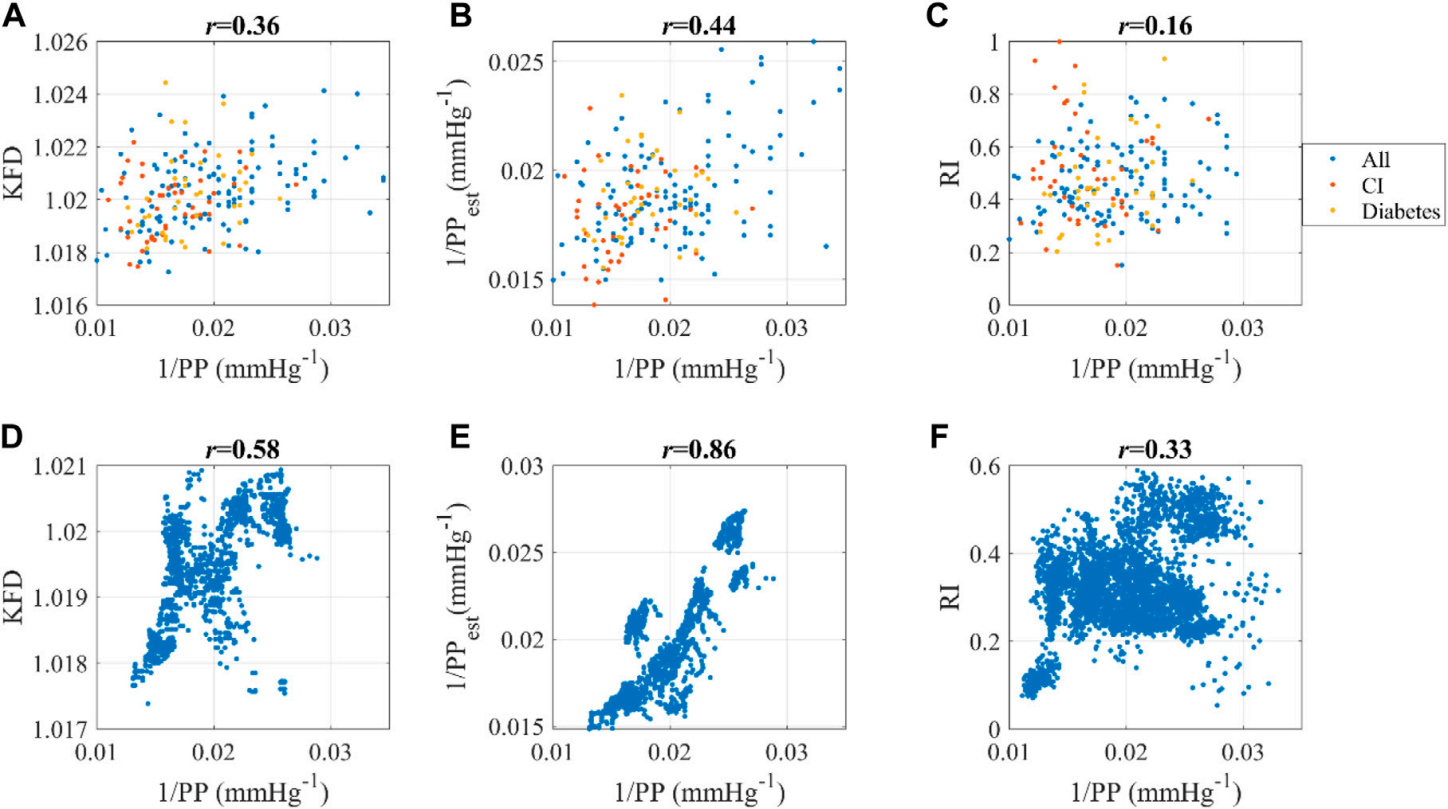
Dataset1: Correlation between **(A)** 1/PP and KFD, **(B)** 1/PP and 1/PP_est_, **(C)** 1/PP and RI. “All” indicates all the data; CI refers to cerebral infarction. Dataset2: Correlation between **(D)** 1/PP and KFD, **(E)** 1/PP and 1/PP_est_, **(F)** 1/PP and RI. Pearson correlation coefficient was used.

The correlation between KFD and PP^−1^ were 0.36 and 0.58 respectively for Dataset1 and Dataset2. In comparison, the best correlations achieved by PPG-morphology-derived indices with PP^−1^ were −0.18 and 0.33, respectively, significantly lower than the proposed KFD method. The detailed results are presented in Table 3. It is worth noting that, the removed outliers are not the same for KFD and PPG-morphology-derived features, leading to a slight mismatch of the used measurements. While AGI displayed a slightly stronger correlation than RI in Dataset1, the difference was not statistically significant, with confidence interval widths of 0.06 for AGI and 0.05 for RI, respectively. Therefore, we opted to depict the correlation of RI and PP^−1^ in Figure 10. The correlation of PPG-morphology-derived features and PP^−1^ also depends on SNR, as expected. By partitioning the data in Dataset1 based on the median SNR value, we observed that measurements with low SNR exhibited a correlation of 0.05 between RI and PP^−1^, whereas measurements with high SNR showed a correlation of 0.19 between RI and PP^−1^. In terms of KFD, the disparity between the various SNR groups was 0.31 and 0.39, indicating a relatively smaller contrast.

**TABLE 3:** Correlations of PPG-derived indices and PP^−1^.

	KFD	PP_est_^-1^	SI_ppg_	RI	AGI
Dataset1	0.36	0.44	−0.14	0.16	−0.18
Dataset2	0.58	0.86	−0.12	0.33	−0.18

The linear combination of ΔV_ppg_m_ and KFD yields 1/PP_est_, which has an even stronger correlation with 1/PP compared to KFD alone, as demonstrated in Figure 10. This observation aligns with the findings from the *in silico* simulation presented in Figure 4. Another interesting finding is that this correlation depends on health status. Dataset1 consists of individuals with cerebral infarction (N = 45) and diabetes (N = 38). Our findings indicate that for these subjects, the correlation coefficients of estimated and measured PP decreased to 0.26, whereas it was 0.49 for both healthy and hypertensive subjects without other known complications, regardless of age. It is worth noting that ΔV_ppg_m_ and KFD were derived independently from the PPG signal, while PP was measured using a separate device. Consequently, this comparison provides a rigorous evaluation of the relationship between these variables, indicating the robustness of *g* (KFD).

While the correlation of AC and its surrogate measure is influenced by noise levels and filtering techniques, which can complicate comparisons between datasets, we believe it remains valuable to compare our results with those from previous publications, as shown in Table 4. Different surrogate measures, such as PP, Age, and PWV, were utilized to assess the correlation of AC with PPG-derived features. Even when examining the same PPG-morphology-derived feature like SI_ppg_, its correlation with the AC surrogate measure could vary significantly from 0.65 to 0.1, depending on the particular cohort and measurement conditions. We hypothesize that noise may be the primary contributor to this phenomenon.

**TABLE 4:** Comparison with previous studies using single-site PPG.

Study	Protocol	AC surrogate measures	PPG-derived features	Correlation (*r*)
Trumpp et al. (2017)	70 patients after surgery (age not reported), camera-based PPG	PP (invasive)	PPG amplitude	0.54
Takazawa et al. (1998)	39 patients (54 ± 11 years), finger PPG	Age	AGI	0.8
Park et al. (2019)	262 women outpatients (38.57 ± 11.64 years), finger PPG	PP (Wrist)	AGI	0.18
Millasseau et al. (2002)	87 asymptomatic subjects (21–68 years; 29 women), finger PPG	cfPWV	SI	0.65
Said et al. (2018)	169 613 individuals (45.8% males; average age 56.8 years old) participating in United Kingdom Biobank, finger PPG, *r* ^2^ was reported	PP	SI	∼0.1
Padilla et al. (2006)	30 healthy subjects (10 female and 20 male, 24–52 years old), finger PPG	baPWV	RI SI	0.32 0.57

cfPWV, Carotid-femoral pulse wave velocity; baPWV, Brachial-ankle pulse wave velocity.

### 3.6 Experimental sensitivity of KFD to hemodynamic parameters

Moreover, consistent with the findings from the *in silico* simulation, the sensitivity of KFD to AC was observed to vary, exhibiting both positive and negative correlations depending on the hemodynamic status and underlying causes of fluctuations. To delve deeper into this phenomenon, we conducted a partial correlation analysis of KFD with SV, PP, and *R* using Dataset2, which consisted of continuous recordings. We then compared these results with the predictions generated by the *in silico* simulation.

The relationship between the resulting sensitivity and hemodynamic status in Dataset2 generally aligned with the *in silico* prediction, with two notable discrepancies, as shown in Table 5. Firstly, measurements with positive ∂KFD/∂R demonstrated lower PP or higher AC, which was not observed in the *in silico* data. Additionally, in Dataset2, the majority of individuals displayed a positive sensitivity to variations in all three parameters: SV, AC, and R. In contrast, the *in silico* data indicated that KFD primarily exhibited a positive sensitivity only when AC was altered. It is important to acknowledge that these discrepancies could be attributed to the distribution of the data. The *in silico* simulation assumes a homogeneous distribution of hemodynamic status, whereas real-world data typically follows a Gaussian distribution and relies on the characteristics of the study population. In the case of our analysis, Dataset2 consisted of a younger and overall healthy population, which contrasts with PWDB that includes virtual subjects ranging from 25 to 75 years old. Therefore, this mismatch is reasonable, considering the actual occurrence probability.

**TABLE 5:** Sensitivity of KFD to PP^−1^, *R*, and SV based on hemodynamic status.

*I*_individual_	Positive *I*_individual_				Negative *I*_individual_			
	N (%)	SV (mL)	PP (mmHg)	*R* (mmHg.s/mL)	N (%)	SV (mL)	PP (mmHg)	*R* (mmHg.s/mL)
$\frac{\partial \text{KFD}}{\partial {\text{PP}}^{-1}}$	70.0	96.8 ± 23.4	48.3 ± 8.3*	1.29 ± 0.31*	30.0	97.6 ± 34.2	53.7 ± 13.5*	1.41 ± 0.51*
$\frac{\partial \text{KFD}}{\partial R}$	87.5	94.9 ± 24.7	48.0 ± 8.7*	1.39 ± 0.40	12.5	99.1 ± 33.6	54.1 ± 13.3*	1.34 ± 0.48
$\frac{\partial \text{KFD}}{\partial \text{SV}}$	82.5	84.8 ± 26.9*	51.4 ± 12.1	1.55 ± 0.49*	17.5	110.4 ± 27.7*	51.6 ± 11.7	1.16 ± 0.26*

N, Percentage of positive or negative *I*_individual_ in real human measurements. * indicate a significant difference between the hemodynamic parameters in the positive or negative *I*_individual_ group. A Student’s t-test with uneven sample sizes was used.

## 4 Discussion

### 4.1 Novelty and main finding

Changes in arterial compliance can indicate the presence of underlying health conditions such as arterial stiffness, atherosclerosis, or hypertension. By monitoring arterial compliance over time, healthcare professionals can gain insights into the progression of these conditions and make informed decisions regarding treatment and intervention strategies (Pannier et al., 2002; Budoff et al., 2021). Although PPG-derived SI_ppg_ may produce consistent results in noiseless virtual human simulations (Hong et al., 2023), it is prone to significant errors under low SNR conditions and is not a robust index in the real world. These findings suggest that while AC information can be transmitted to peripheral sites with relatively high fidelity, the main challenge lies in preserving this information under low SNR conditions.

Our study presents compelling evidence for using KFD instead of SI_ppg_ for AC assessment, particularly due to KFD’s noise resistance properties. In addition, KFD has an exponential association with the ground truth of FD, which renders it particularly apt for signals displaying exponential characteristics. Given the known exponential relationship between AC and blood vessel radius *r* (AC∝exp (-k∙r)), KFD appears to be a particularly suitable choice compared to other FD measures. Virtual human studies provided an advantageous platform for thorough comparison. By controlling hemodynamic distribution, noise levels, and utilizing transparent simulation parameters, we could isolate the factors affecting temporal patterns and generate more interpretable results. In simulated scenarios, we discovered that SI_ppg_’s correlation with AC declines sharply when exposed to high noise levels, making it unsuitable for ambulatory testing. The same trend was observed for other PPG-morphology-derived AC measures like AGI and RI. Conversely, KFD maintains a strong correlation with AC even in noisy environments, thanks to its resilience against such disturbances. This characteristic positions KFD as a superior alternative to SI_ppg_. Furthermore, KFD is easy to use. Even in scenarios with low SNR, KFD remains robust with knowledge-based multi-Gaussian filtering. This is because KFD deciphers the AC-related information encoded in the temporal patterns. A few minor distortions do not significantly impact the overall shape or fractal dimension.

KFD is originally a mathematical concept. In order to obtain a comprehensive understanding of the physiological meaning of PPG’s KFD, we conducted a thorough analysis by comparing three *in silico* models and *in vivo* data. Our investigation revealed that the correlation between KFD and AC likely arises from the variation of arterial compliance throughout the cardiac cycle, which is further enhanced by the tree-like structure of the arterial system.

To validate our findings from *in silico* databases, we utilized two experimental datasets. In the case of real human data, we used PP as an alternative measure for arterial compliance (AC), as suggested in previous literature (Bortel et al., 2001) and supported by our own analysis, which showed a high correlation of PP^−1^ and AC (*r* = 0.78). In Dataset2, which consisted of 40 young and healthy subjects, we observed a strong correlation between KFD and PP^−1^ (*r* = 0.58). This correlation was slightly lower than the corresponding correlation in the *in silico* simulation (PWDB, *r* = 0.65). However, it increased to 0.86 after incorporating ΔV_ppg_m_, which was higher than the corresponding correlation in the *in silico* simulation (PWDB, *r* = 0.72). This unexpected observation can be attributed to the threshold effect provided by ΔV_ppg_m_, which removed subjects with poor perfusion. The remaining data may have had a higher center-peripheral correlation. Subjects with high BMI also experience a loss of correlation between KFD and AC. This could be attributed to excessive pressure on the finger or difficulties in capturing the pulse of the vascular bed. In contrast, the best correlations attained by PPG morphology-derived features were −0.18 and 0.33, achieved by AGI and RI, respectively. RI exhibited better performance in Dataset2, likely attributed to its stronger correlation with PP^−1^ in the high PP region (>70 mmHg).

In Dataset1, which comprised 219 older subjects with various health conditions, the estimated and measured PP^−1^ correlation was weaker (*r* = 0.44), which only improved to 0.49 for a relatively healthier subpopulation without cerebral infarction or diabetes. This difference between two datasets can be attributed to variations in the measurement protocols used. In Dataset2, BP was measured at the fingertip and then reconstructed into brachial BP, which may encourage a stronger correlation with finger PPG-derived KFD. In addition, Dataset1 consisted of short measurements with reference values obtained from the opposite arm, introducing a larger uncertainty in the reference. Furthermore, the subjects in Dataset1 were significantly older compared to those in Dataset2 and the signal is considerably noisier. Health status is another significant confounding factor in this analysis, particularly when assessing AC or PP of subjects with cerebral infarction or diabetes. The lower correlation in subjects with these health conditions may arise due to the potential alteration of their hemodynamic status as a result of the disease, leading to distortion to features or fluctuations. Although posture differences may also act as confounding factors, additional investigation is required to determine the extent of their influence on the association between KFD and AC. No sex difference was found.

An interesting observation is that the sensitivity of KFD varies depending on the hemodynamic status of the subjects. Specifically, KFD demonstrates a predominantly positive sensitivity to AC variation, indicating consistent tracking ability despite regions showing diminished sensitivity. The sensitivity of KFD to SV or *R* can exhibit both positive and negative values, which can interfere with AC estimation. While KFD alone may not be suitable as an intra-subject AC tracking index, when combined with morphological features and other complexity indices, it provides more robust information about the hemodynamic status. This is evident in their ability to exhibit distinct patterns of positive or negative sensitivity in response to known stimuli such as changes in posture, mental arithmetic, and exercise. Previous research has demonstrated the utility of PPG feature interactions in boosting arterial stiffness estimation precision (Chen et al., 2024). In our context, such patterns can be utilized to minimize influences from confounding factors and isolate the AC-induced changes, thereby enhancing AC estimation, reducing uncertainties caused by signal noise and instability.

In summary, these findings provide valuable insights into the application of KFD for arterial compliance assessment.

### 4.2 Limitation

There are several limitations in this study. Firstly, although we have supportive evidence from *in silico* simulation, the experiment data used did not include direct measurements of AC. Instead, an intermediate measure, the pulse pressure, was utilized. While this surrogate measure provided valuable insights and aligned with the *in silico* simulation, it is essential to conduct future experiments to validate and confirm the results. Future studies should aim to incorporate more rigorous measures, such as aortic pulse wave velocity (aoPWV), or invasive measurements, to estimate AC accurately.

Secondly, the 1D model for the *in silico* simulation employed in this study, although capturing vascular tree information, is not a closed-loop model. It represents stable hemodynamics and generates simulation data for one single cardiac cycle. However, in reality, cardiac output and hemodynamic parameters undergo continuous changes at varying rates, influenced by metabolic needs and the pulmonary circulation (Westerhof et al., 2019). These issues should be considered in future research.

Moreover, the study did not include heart rate variation and other long-term autocorrelation measures, such as the autocorrelation of beat-to-beat CO and ΔV_ppg_, to investigate long-term fractality (Sviridova and Sakai, 2015; Xing et al., 2023b). Fortunately, KFD only requires a very short segment of data, namely, a cardiac cycle. However, other fractal dimensions, such as Higuchi fractal dimension and Permutation entropy, which have proven useful in cardiovascular and metabolic disease monitoring, require longer recording times (Wei et al., 2020; Xing et al., 2023b). Their correlation with PP should also be investigated and compared with KFD. To explore the theoretical basis of these measures, longer simulations and more comprehensive models should be developed.

Theoretically, we should have more than 80% of the AC information transmitted from the center to the periphery, assuming a noiseless SI_ppg_ performance (Hong et al., 2023). *In silico* studies could help us identify where the information is lost. While the application of KFD has addressed the issue of noise, there are still other factors that need to be considered and remedied. These factors include the cardiac blood flow waveform, turbulence, partial blockage of the vasculature, and auto-regulation, among others (Westerhof et al., 2019).

Our study exclusively focused on applying KFD to transmissive PPG, which has a well-established theoretical foundation. For alternative PPG modalities, like remote PPG acquired via cameras, the unique experimental setups and signal generation processes must be taken into account, with the possibility of adapting insights gained from transmissive PPG after appropriate modifications (Kamshilin et al., 2015; Moço et al., 2018).

## 5 Conclusion

Katz’s fractal dimension of PPG displays a robust correlation with arterial compliance. Possessing exceptional noise resistance, it reliably estimates AC even in demanding scenarios. The study demonstrates that this heightened efficacy stems from KFD’s capacity to encapsulate signal complexity arising from compliance fluctuations across a cardiac cycle and intricate vascular structures, diminishing the need for specific characteristic points. This investigation results in an innovative method for ongoing, wearable monitoring of arterial compliance through PPG signals, offering potential for improved cardiovascular health evaluation.

## Data Availability

The original contributions presented in the study are included in the article/supplementary material, further inquiries can be directed to the corresponding authors.

## Appendix

In this study, we utilized two lumped WK4 models to generate simplified versions of PWDB. These 0D models incorporated central compliance (*C*_1_), peripheral compliance (*C*_2_), peripheral resistance (*R*), and inertance (*L*) as its key components (Figure 1). Since PWDB did not explicitly provide an inertance value and *L* is inversely correlated with arterial cross-sectional area, we hypothesized that *L* is linearly correlated with SI_ppg_ (Stergiopulos et al., 1999; Wang et al., 2017). Additionally, we integrated CO, heart rate, duration of left ventricle ejection and the peak occurring time during systole from PWDB into the 0D PPG simulations. These parameters help to build the cardiac flow waveform *q*_in_(t), as shown in Figures 1A, B. For each virtual subject in PWDB, we used the transfer function from finger BP to PPG as described in (Charlton et al., 2018). This transfer function was then used to map the peripheral BP waveform (*p*_p_(t)) generated by the 0D model to the corresponding PPG waveform.

For the lumped WK4 model with fixed *C*_1_(t), equation 1 could be solved using the Runge-Kutta (4,5) formula (ODE45). The step-to-step solving procedure was demonstrated by Wang *et al.* (Wang et al., 2017). For the lumped WK4 model with time-dependent *C*_1_(t), we adopted the definition of *C*_1_ proposed by Langewouters *et al.* (Langewouters et al., 1984), as shown in Eq. A.1. This definition allows the *C*_1_ to vary with pressure and simplifies the numerical solution to the time-dependent WK4 model equations, as demonstrated in our previous research (Xing et al., 2023b).

$$C_{1}\left(t\right)=\frac{A_{m}l}{\pi P_{1}\left[1+{\left(\left(p_{c}\left(t\right)-P_{0}\right)/P_{1}\right)}^{2}\right]}$$
(A.1)

*A*_m_ is the maximum cross-sectional area of the aorta, *l* is the unit length and *P*_0_ is the transmural pressure when compliance reached its maximum. *P*_1_ represents the steepness of the compliance rise. Since the *C*_1_ profile was determined by three variables: *A*_m_ *l*, *P*_0_ and *P*_1_, we used the Young’s modulus profile in PWDB to estimate *A*_m_ *l*, *P*_0_ and *P*_1_ and produce a matching *C*_1_ profile. By defining these variables, we can express all the unknowns in Eq. 2 as functions of *p*_p_(t). Although this equation is more complex compared to the one with a fixed *C*_1_, it is still solvable using the Runge-Kutta (4,5) formula (ODE45). This approach allows us to obtain a time-dependent *p*_p_(t) waveform. The detailed derivation procedure was shown in our previous publication (Xing et al., 2023b).

## References

[B1] Banerjee R. Ghose A. Choudhury A. D. Sinha A. Pal A. (2015). “Noise cleaning and Gaussian modeling of smart phone photoplethysmogram to improve blood pressure estimation,” in 2015 IEEE International Conference on Acoustics, Speech and Signal Processing (ICASSP), South Brisbane, QLD, Australia, 19-24 April 2015, 967–971. 10.1109/icassp.2015.7178113

[B2] Bishop S. M. Ercole A. (2018). “Multi-scale peak and Trough Detection optimised for periodic and quasi-periodic neuroscience data,” in Intracranial pressure and neuromonitoring XVI. Editor Heldt T. (Springer International Publishing), 189–195. 10.1007/978-3-319-65798-1_39 29492559

[B3] Bortel L. M. A. B. V. Struijker-Boudier H. a.J. Safar M. E. (2001). Pulse pressure, arterial stiffness, and drug treatment of hypertension. Hypertension 38, 914–921. 10.1161/hy1001.095773 11641309

[B4] Budoff M. J. Alpert B. Chirinos J. A. Fernhall B. Hamburg N. Kario K. (2021). Clinical applications measuring arterial stiffness: an expert consensus for the application of cardio-ankle vascular index. Am. J. Hypertens. 35, 441–453. 10.1093/ajh/hpab178 PMC9088840 34791038

[B5] Carlson C. Turpin V. R. Suliman A. Ade C. Warren S. Thompson D. E. (2020a). Bed-based ballistocardiography: dataset and ability to track cardiovascular parameters. Sensors (Basel) 21, 156. 10.3390/s21010156 33383739 PMC7795624

[B6] Carlson C. T. V. R. Suliman A. Ade C. Warren S. Thompson D. E. (2020b) Bed-based ballistocardiography dataset. 10.3390/s21010156 PMC7795624 33383739

[B7] Charlton P. H. Celka P. Farukh B. Chowienczyk P. Alastruey J. (2018). Assessing mental stress from the photoplethysmogram: a numerical study. Physiol. Meas. 39, 054001. 10.1088/1361-6579/aabe6a 29658894 PMC5964362

[B8] Charlton P. H. Harana J. M. Vennin S. Li Y. Chowienczyk P. Alastruey J. (2019). Modeling arterial pulse waves in healthy aging: a database for *in silico* evaluation of hemodynamics and pulse wave indexes. Am. J. Physiology-Heart Circulatory Physiology 317, H1062–H1085. 10.1152/ajpheart.00218.2019 PMC6879924 31442381

[B9] Charlton P. H. Kotzen K. Mejía-Mejía E. Aston P. J. Budidha K. Mant J. (2022). Detecting beats in the photoplethysmogram: benchmarking open-source algorithms. Physiol. Meas. 43, 085007. 10.1088/1361-6579/ac826d PMC9393905 35853440

[B10] Charlton P. H. Paliakaitė B. Pilt K. Bachler M. Zanelli S. Kulin D. (2021). Assessing hemodynamics from the photoplethysmogram to gain insights into vascular age: a review from VascAgeNet. Am. J. Physiology-Heart Circulatory Physiology 322, H493–H522. 10.1152/ajpheart.00392.2021 PMC8917928 34951543

[B11] Chen Y. Yang X. Song R. Liu X. Zhang J. (2024). Predicting arterial stiffness from single-channel photoplethysmography signal: a feature interaction-based approach. IEEE J. Biomed. Health Inf., 1–12. 10.1109/jbhi.2024.3383234 38551821

[B12] Couceiro R. Carvalho P. Paiva R. P. Henriques J. Quintal I. Antunes M. (2015). Assessment of cardiovascular function from multi-Gaussian fitting of a finger photoplethysmogram. Physiol. Meas. 36, 1801–1825. 10.1088/0967-3334/36/9/1801 26235798

[B13] Elgendi M. (2012). On the analysis of fingertip photoplethysmogram signals. Curr. Cardiol. Rev. 8, 14–25. 10.2174/157340312801215782 22845812 PMC3394104

[B14] Elgendi M. (2016). Optimal signal quality index for photoplethysmogram signals. Bioeng. (Basel) 3, 21. 10.3390/bioengineering3040021 PMC5597264 28952584

[B15] Esteller R. Vachtsevanos G. Echauz J. Litt B. (2001). A comparison of waveform fractal dimension algorithms. IEEE Trans. Circuits Syst. I Fundam. Theory Appl. 48, 177–183. 10.1109/81.904882

[B16] Hong J. Nandi M. Charlton P. H. Alastruey J. (2023). Noninvasive hemodynamic indices of vascular aging: an *in silico* assessment. Am. J. Physiology-Heart Circulatory Physiology 325, H1290–H1303. 10.1152/ajpheart.00454.2023 PMC10908403 37737734

[B17] Kamshilin A. A. Nippolainen E. Sidorov I. S. Vasilev P. V. Erofeev N. P. Podolian N. P. (2015). A new look at the essence of the imaging photoplethysmography. Sci. Rep. 5, 10494. 10.1038/srep10494 25994481 PMC4440202

[B18] Khalid S. G. Liu H. Zia T. Zhang J. Chen F. Zheng D. (2020). Cuffless blood pressure estimation using single channel photoplethysmography: a two-step method. IEEE Access 8, 58146–58154. 10.1109/access.2020.2981903

[B19] Khodabakhshi M. B. Eslamyeh N. Sadredini S. Z. Ghamari M. (2022). Cuffless blood pressure estimation using chaotic features of photoplethysmograms and parallel convolutional neural network. Comput. Methods Programs Biomed. 226, 107131. 10.1016/j.cmpb.2022.107131 36137326

[B20] Langewouters G. J. Wesseling K. H. Goedhard W. J. A. (1984). The static elastic properties of 45 human thoracic and 20 abdominal aortas *in vitro* and the parameters of a new model. J. Biomechanics 17, 425–435. 10.1016/0021-9290(84)90034-4 6480618

[B21] Laurent S. Cockcroft J. Van Bortel L. Boutouyrie P. Giannattasio C. Hayoz D. (2006). Expert consensus document on arterial stiffness: methodological issues and clinical applications. Eur. Heart J. 27, 2588–2605. 10.1093/eurheartj/ehl254 17000623

[B22] Lee H.-C. Park Y. Yoon S. B. Yang S. M. Park D. Jung C.-W. (2022). VitalDB, a high-fidelity multi-parameter vital signs database in surgical patients. Sci. Data 9, 279. 10.1038/s41597-022-01411-5 35676300 PMC9178032

[B23] Li S. Liu L. Wu J. Tang B. Li D. (2018). Comparison and noise suppression of the transmitted and reflected photoplethysmography signals. BioMed Res. Int. 2018, 4523593. 10.1155/2018/4523593 30356404 PMC6178150

[B24] Liang Y. Chen Z. Liu G. Elgendi M. (2018). A new, short-recorded photoplethysmogram dataset for blood pressure monitoring in China. Sci. Data 5, 180020. 10.1038/sdata.2018.20 29485624 PMC5827692

[B25] Mancia G. Fagard R. Narkiewicz K. Redón J. Zanchetti A. Böhm M. (2013). Guidelines for the management of hypertension and target organ damage: reply. J. Hypertens. 31, 2464–2465. 10.1097/HJH.0000000000000006 24220597

[B26] Mccool P. Altmann Y. Perperidis A. Mclaughlin S. (2016). “Robust Markov Random Field outlier detection and removal in subsampled images,” in 2016 IEEE Statistical Signal Processing Workshop (SSP), Palma de Mallorca, Spain, 26-29 June 2016, 1–5. 10.1109/ssp.2016.7551766

[B27] Mejía-Mejía E. May J. M. Elgendi M. Kyriacou P. A. (2021). Classification of blood pressure in critically ill patients using photoplethysmography and machine learning. Comput. Methods Programs Biomed. 208, 106222. 10.1016/j.cmpb.2021.106222 34166851

[B28] Millasseau S. C. Kelly R. P. Ritter J. M. Chowienczyk P. J. (2002). Determination of age-related increases in large artery stiffness by digital pulse contour analysis. Clin. Sci. (Lond) 103, 371–377. 10.1042/cs1030371 12241535

[B29] Moço A. V. Stuijk S. De Haan G. (2018). New insights into the origin of remote PPG signals in visible light and infrared. Sci. Rep. 8, 8501. 10.1038/s41598-018-26068-2 29855610 PMC5981460

[B30] Monge-Álvarez J. (2024) Higuchi and Katz fractal dimension measures. MATLAB Central File Exchange. Available at: https://www.mathworks.com/matlabcentral/fileexchange/50290-higuchi-and-katz-fractal-dimension-measures.

[B31] Niiranen T. J. Kalesan B. Mitchell G. F. Vasan R. S. (2019). Relative contributions of pulse pressure and arterial stiffness to cardiovascular disease. Hypertension 73, 712–717. 10.1161/HYPERTENSIONAHA.118.12289 30661478 PMC6374179

[B32] Orphanidou C. Bonnici T. Charlton P. Clifton D. Vallance D. Tarassenko L. (2015). Signal-quality indices for the electrocardiogram and photoplethysmogram: derivation and applications to wireless monitoring. IEEE J. Biomed. Health Inf. 19, 832–838. 10.1109/JBHI.2014.2338351 25069129

[B33] Padilla J. M. Berjano E. J. Saiz J. Facila L. Diaz P. Merce S. (2006). “Assessment of relationships between blood pressure, pulse wave velocity and digital volume pulse,” in 2006 Computers in Cardiology, Valencia, Spain, 17-20 September 2006, 893–896.

[B34] Pannier B. M. Avolio A. P. Hoeks A. Mancia G. Takazawa K. (2002). Methods and devices for measuring arterial compliance in humans. Am. J. Hypertens. 15, 743–753. 10.1016/s0895-7061(02)02962-x 12160200

[B35] Park Y. J. Lee J. M. Kwon S. H. (2019). Association of the second derivative of photoplethysmogram with age, hemodynamic, autonomic, adiposity, and emotional factors. Med. Baltim. 98, e18091. 10.1097/MD.0000000000018091 PMC6882620 31764845

[B36] Pereira T. Correia C. Cardoso J. (2015). Novel methods for pulse wave velocity measurement. J. Med. Biol. Eng. 35, 555–565. 10.1007/s40846-015-0086-8 26500469 PMC4609308

[B37] Pereira T. Santos I. Oliveira T. Vaz P. Correia T. Pereira T. (2013). Characterization of optical system for hemodynamic multi-parameter assessment. Cardiovasc. Eng. Technol. 4, 87–97. 10.1007/s13239-013-0125-y

[B38] Radha M. De Groot K. Rajani N. Wong C. C. P. Kobold N. Vos V. (2019). Estimating blood pressure trends and the nocturnal dip from photoplethysmography. Physiol. Meas. 40, 025006. 10.1088/1361-6579/ab030e 30699397

[B39] Redheuil A. Yu W. C. Wu C. O. Mousseaux E. De Cesare A. Yan R. (2010). Reduced ascending aortic strain and distensibility: earliest manifestations of vascular aging in humans. Hypertension 55, 319–326. 10.1161/HYPERTENSIONAHA.109.141275 20065154 PMC3035625

[B40] Said M. A. Eppinga R. N. Lipsic E. Verweij N. Van Der Harst P. (2018). Relationship of arterial stiffness index and pulse pressure with cardiovascular disease and mortality. J. Am. Heart Assoc. 7, e007621. 10.1161/JAHA.117.007621 29358193 PMC5850166

[B41] Schlesinger O. Vigderhouse N. Moshe Y. Eytan D. (2020). Estimation and tracking of blood pressure using routinely acquired photoplethysmographic signals and deep neural networks. Crit. Care Explor. 2, e0095. 10.1097/CCE.0000000000000095 32426737 PMC7188414

[B42] Segers P. Rietzschel E. R. De Buyzere M. L. Stergiopulos N. Westerhof N. Van Bortel L. M. (2008). Three- and four-element Windkessel models: assessment of their fitting performance in a large cohort of healthy middle-aged individuals. Proc. Institution Mech. Eng. Part H J. Eng. Med. 222, 417–428. 10.1243/09544119JEIM287 18595354

[B43] Shin H. (2022). Deep convolutional neural network-based signal quality assessment for photoplethysmogram. Comput. Biol. Med. 145, 105430. 10.1016/j.compbiomed.2022.105430 35339844

[B44] Stergiopulos N. Westerhof B. E. Westerhof N. (1999). Total arterial inertance as the fourth element of the windkessel model. Am. J. Physiology-Heart Circulatory Physiology 276, H81–H88. 10.1152/ajpheart.1999.276.1.H81 9887020

[B45] Sviridova N. Sakai K. (2015). Human photoplethysmogram: new insight into chaotic characteristics. Chaos, Solit. Fractals 77, 53–63. 10.1016/j.chaos.2015.05.005

[B46] Sviridova N. Zhao T. Aihara K. Nakamura K. Nakano A. (2018). Photoplethysmogram at green light: where does chaos arise from? Chaos, Solit. Fractals 116, 157–165. 10.1016/j.chaos.2018.09.016

[B47] Takazawa K. Tanaka N. Fujita M. Matsuoka O. Saiki T. Aikawa M. (1998). Assessment of vasoactive agents and vascular aging by the second derivative of photoplethysmogram waveform. Hypertension 32, 365–370. 10.1161/01.hyp.32.2.365 9719069

[B48] Tang Q. Chen Z. Allen J. Alian A. Menon C. Ward R. (2020). PPGSynth: an innovative toolbox for synthesizing regular and irregular photoplethysmography waveforms. Front. Med. 7, 597774. 10.3389/fmed.2020.597774 PMC7668389 33224967

[B49] Trumpp A. Rasche S. Wedekind D. Rudolf M. Malberg H. Matschke K. (2017). Relation between pulse pressure and the pulsation strength in camera-based photoplethysmograms. Curr. Dir. Biomed. Eng. 3, 489–492. 10.1515/cdbme-2017-0184

[B50] Vlachopoulos C. Aznaouridis K. Stefanadis C. (2010). Prediction of cardiovascular events and all-cause mortality with arterial stiffness: a systematic review and meta-analysis. J. Am. Coll. Cardiol. 55, 1318–1327. 10.1016/j.jacc.2009.10.061 20338492

[B51] Voges I. Jerosch-Herold M. Hedderich J. Pardun E. Hart C. Gabbert D. D. (2012). Normal values of aortic dimensions, distensibility, and pulse wave velocity in children and young adults: a cross-sectional study. J. Cardiovasc. Magnetic Reson. 14, 77. 10.1186/1532-429X-14-77 PMC3514112 23151055

[B52] Wang L. Xu L. Zhou S. Wang H. Yao Y. Hao L. (2017). Design and implementation of a pulse wave generator based on Windkessel model using field programmable gate array technology. Biomed. Signal Process. Control 36, 93–101. 10.1016/j.bspc.2017.03.008

[B53] Wang S. Wu D. Li G. Song X. Qiao A. Li R. (2022). A machine learning strategy for fast prediction of cardiac function based on peripheral pulse wave. Comput. Methods Programs Biomed. 216, 106664. 10.1016/j.cmpb.2022.106664 35104684

[B54] Wei H. C. Ta N. Hu W. R. Wang S. Y. Xiao M. X. Tang X. J. (2020). Percussion entropy analysis of synchronized ECG and PPG signals as a prognostic indicator for future peripheral neuropathy in type 2 diabetic subjects. Diagn. (Basel) 10, 32. 10.3390/diagnostics10010032 PMC7168256 31936481

[B55] Westerhof N. Stergiopulos N. Noble M. Westerhof B. (2019) Snapshots of hemodynamics: an aid for clinical research and graduate education. Springer.

[B56] Xing X. Dong W.-F. Xiao R. Song M. Jiang C. (2023a). Analysis of the chaotic component of photoplethysmography and its association with hemodynamic parameters. Entropy 25, 1582. 10.3390/e25121582 38136462 PMC10742563

[B57] Xing X. Huang R. Hao L. Jiang C. Dong W.-F. (2023b). Temporal complexity in photoplethysmography and its influence on blood pressure. Front. Physiology 14, 1187561. 10.3389/fphys.2023.1187561 PMC10513039 37745247

